# Enhanced Locomotion Efficiency of a Bio-inspired Walking Robot using Contact Surfaces with Frictional Anisotropy

**DOI:** 10.1038/srep39455

**Published:** 2016-12-23

**Authors:** Poramate Manoonpong, Dennis Petersen, Alexander Kovalev, Florentin Wörgötter, Stanislav N. Gorb, Marlene Spinner, Lars Heepe

**Affiliations:** 1 Embodied AI and Neurorobotics Lab, Centre for BioRobotics, The Mærsk Mc-Kinney Møller Institute, University of Southern Denmark, Odense M, DK-5230, Denmark; 2 Bernstein Center for Computational Neuroscience (BCCN), The Third Institute of Physics, Georg-August-Universität Göttingen, Göttingen, D-37077, Germany; 3 Department of Functional Morphology and Biomechanics, Zoological Institute, Kiel University, Kiel, D-24118, Germany; 4 Mads Clausen Institute, University of Southern Denmark, Sønderborg, DK-6400, Denmark

## Abstract

Based on the principles of morphological computation, we propose a novel approach that exploits the interaction between a passive anisotropic scale-like material (e.g., shark skin) and a non-smooth substrate to enhance locomotion efficiency of a robot walking on inclines. Real robot experiments show that passive tribologically-enhanced surfaces of the robot belly or foot allow the robot to grip on specific surfaces and move effectively with reduced energy consumption. Supplementing the robot experiments, we investigated tribological properties of the shark skin as well as its mechanical stability. It shows high frictional anisotropy due to an array of sloped denticles. The orientation of the denticles to the underlying collagenous material also strongly influences their mechanical interlocking with the substrate. This study not only opens up a new way of achieving energy-efficient legged robot locomotion but also provides a better understanding of the functionalities and mechanical properties of anisotropic surfaces. That understanding will assist developing new types of material for other real-world applications.

Animals can traverse difficult terrains (e.g., inclined and uneven substrates) as well as adhere to surfaces in an energy-efficient way. Biological studies reveal that the attachment devices they use are one of the key features supporting these achievements^1^,^2^,^3^,^4^,^5^. These attachment devices can provide optimal friction for forward motion, protect animals from slipping on a surface, and even allow them grip surfaces firmly. From this point of view, several works have investigated biological materials^6^,^7^,^8^,^9^,^10^ and used them as an inspiration to develop materials^11^,^12^ for robotic applications^13^,^14^,^15^,^16^,^17^.

From a robotic point of view, there are two main ways to allow legged robots to traverse difficult terrains including inclined ones: (1) different general control approaches and (2) special robot structures and materials (known as morphological computation^18^,^19^) with specific control strategies. For example, based on the first option, Steingrube *et al*.^20^ developed adaptive neural control to enable a six-legged robot to learn to find an appropriate gait for walking up a slope. Komatsua *et al*.^21^ proposed a control technique to achieve the optimal slope-walking motion for a four-legged robot. Following the second option, Kim *et al*.^15^ developed gecko-inspired adhesive materials and implemented them on a four-legged robot. Using the adhesive materials with a specific control mechanism for peeling toe pads of the surface allows the robot to climb up a smooth wall. A similar strategy has been also applied to a leg-wheel hybrid robot^17^. Voigt *et al*.^14^ used foamy rubber materials as a gripper of the small robot Ratnic, with specific gripper movement control, for climbing on a pipe.

These robots operate mostly on glass, smooth surfaces, or surfaces with low roughness. Extending the operational range to other surfaces (like carpets or other felt-like or rough/dusty substrates), Spenko *et al*.^22^ developed special body and leg structures with compliant feet and embedded microspines^23^ for the hexapod robot RiSE. The robot uses specific climbing control with force feedback to generate proper leg movements for climbing up different rough surfaces. Palmer *et al*.^24^ developed so-called Distributed Inward Gripping (DIG) with torsion springs (i.e., passive compliance) at the legs of a six-legged robot. Using this mechanism with specific leg movement control, the robot can generate adhesive forces and walk vertically on a mesh screen. Bretl^25^ developed a four-legged robot with single peg legs wrapped in high-friction rubber and used multi-step motion planning control to enable the robot to climb vertical rock. While all these approaches show impressive results, they require special control, structure, and material designs to deal with rough surfaces.

Locomotion efficiency on rough surfaces is nontrivial; it can, however, be achieved or improved by employing the concepts of frictional anisotropy and mechanical interlocking between surfaces at the microscale. In principle, strong mechanical interlocking in one direction will allow a robot to grip the surface, thereby preventing it from slipping or sliding backward, while almost no mechanical interlocking in another direction will allow it to easily release itself from the surface while moving forward. On the other hand, having strong mechanical interlocking in both directions (i.e., frictional isotropy) will also allow the robot to grip to the surface but it will have difficulty releasing itself from the surface. Based on these concepts of frictional anisotropy and mechanical interlocking, Marvi *et al*.^26^ developed active scales and their control to generate the frictional anisotropy for the snake-inspired robot Scalybot; thereby allowing it to climb inclines up to 45°. Instead of using active scales as Scalybot, we use here a passive anisotropic scale-like material (shark skin) to enhance the efficiency of legged robot locomotion on inclines. In fact, shark skin was already used in ancient times by fishermen who made their shoes out of this material^27^, potentially to enhance their grip on the wet wooden deck of their ships. Moreover, shark skin was also used in various tools such as in wooden rasps^27^, in grips and sheath of swords, and also to sand wood and ceramics^28^. This highlights the astonishing material properties of shark skin that humans have been aware of for centuries. Our work here is also inspired by this tradition.

For our robot experiments, standard walking patterns are employed to compare the locomotion efficiency of an existing six-legged walking robot, with and without shark skin, on a surface covered by carpet or other felt-like or rough solid substrates. We use the specific resistance also known as the cost of transport (COT)^29^ to demonstrate the energy-efficiency of locomotion. Supplementing the robot experiments, we also systematically investigated the tribological and mechanical properties of shark skin. Particularly, we seek to investigate the effects of sliding direction, normal load, and substrate roughness on the friction behavior as well as its mechanical stability. Thus, this study contributes not only towards energy-efficient walking robots but also to a better understanding of the functionalities and mechanical properties of shark skin, which may guide the development of a new bio-inspired anisotropic scale-like material for future biomimetic applications.

## Results

### Exploiting a passive anisotropic material for enhanced mechanical adhesion and locomotion efficiency in a bio-inspired walking robot

Biological materials, such as shark skin, have an interesting morphology and surface microstructure. On the upper surface of the skin, there is an array of sloped denticles (tooth-like structures), called placoid denticles. From the structure of the denticles, a strong mechanical interlocking is expected on rough substrates while sliding in against the direction of the denticles - the rostral direction. For sliding along the denticles, the caudal direction, low friction is expected. Thus, the morphlogical features of shark skin generate pronounced frictional anisotropy. This frictional anisotropy of shark skin has been used for making a polishing material^27^,^30^, for shoes of fishermen^27^, and in handles and sheath of swords^28^. However, so far it has not been used for robotic implications.

**Figure 1:**
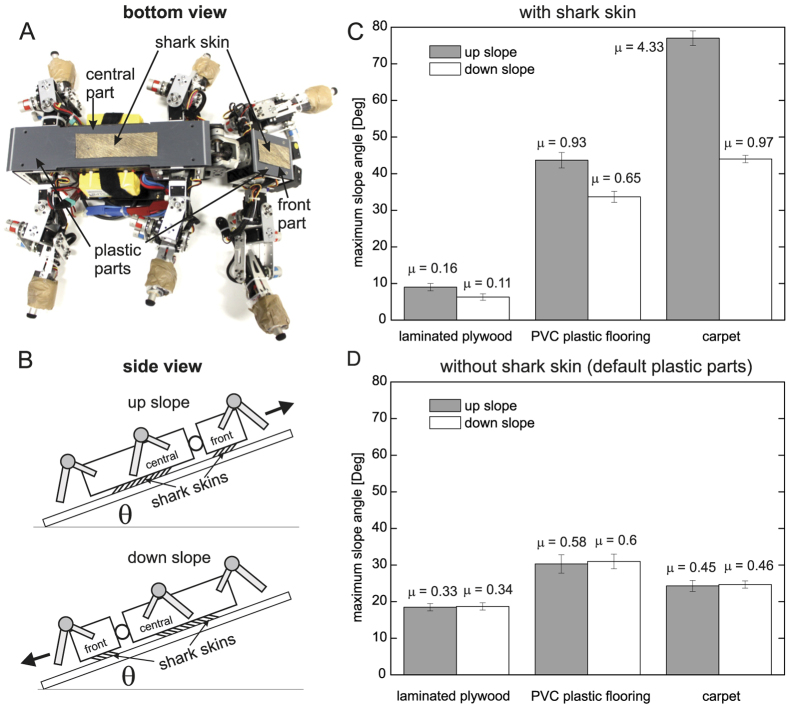
Testing grip of AMOS, with and without shark skin, on different surfaces. (**A**) Two pieces of shark skin installed at the front and central parts of the hexapod walking robot AMOS. The shark skin at the front part has a size of 4 cm wide and 7 cm long while another one at the central part is 4 cm wide and 12 cm long. (**B**) Diagrams showing the static experiments. (**C**), (**D**) A comparison of maximum slope angles before AMOS, with and without shark skin, started to slip on three different surfaces. Average static friction coefficients *μ* between shark skin and the surface for all tests are calculated from tan h(*θ*_*max*_) and depicted on top of the columns. We performed ten runs for each surface. The error bars represent standard deviation.

In the present paper, we show how the frictional anisotropy of shark skin can be exploited to enhance grip and locomotion of a bio-inspired walking robot. We used our hexapod walking robot AMOS with weight of 56.84N as a testbed (see the Methods section for the detail of AMOS). Figure 1A presents the preparation for investigation of robot mechanical adhesion and locomotion where we installed two pieces of dry shark skin on the front and central parts of the belly. Figure 1B shows two setups for the adhesion tests. Figure 1C and D show a comparison of the tests on three different surfaces. We placed the robot, with and without shark skin, on top of a laminated plywood board (surface 1), the plywood board covered by PVC plastic flooring (surface 2), and the plywood board covered by carpet (surface 3).

We gradually increased the angle of the incline until the robot, with and without shark skin, started to slip. We then measured the maximum angle right before slipping occurred. During the tests, the robot legs were fixed to stay above the ground, to ensure that only body parts made contact with the ground. The results show that the shark skin exhibited strong frictional anisotropy, which allowed the robot to grip a rough surface, like carpet, strongly in the rostral direction but with a weaker grip in the caudal direction. Lower grip was observed on smooth and slightly rough surfaces, here laminated plywood board and PVC plastic flooring, respectively. In contrast, the robot without shark skin (i.e., only default plastic body parts with frictional isotropy) showed similar maximum slope angles on the three surfaces in both directions. We encourage the reader to also see Supplementary Movie 1 for another adhesion test. We also tested seal skin as another anisotropic biological material. The results were similar to shark skin but less pronounced (see supplementary information).

**Figure 2:**
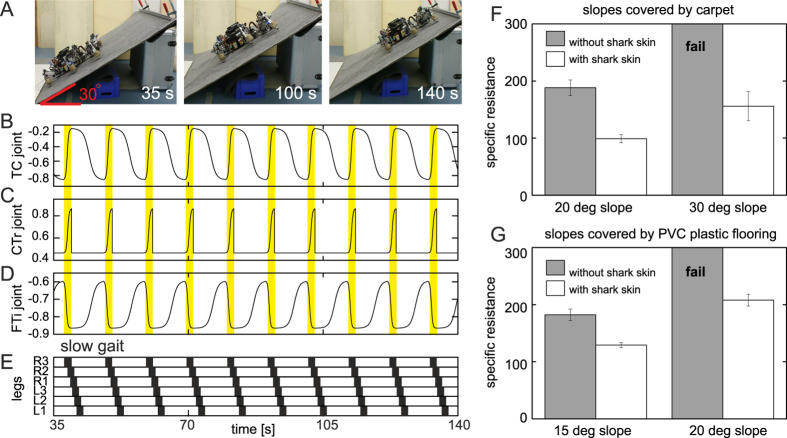
Testing locomotion of AMOS, with and without shark skin on its belly, on different slopes with different surfaces. (**A**) The setup of our dynamic experiments and snap shots of walking up a 30° slope covered by carpet. **(B**,**C**,**D)** The thoraco-coxal (TC-), coxo-trochanteral (CTr-), and femoro-tibial (FTi-) joint angles of the right hind leg (R3, see also Fig. 3A) during walking up the slope. The TC-joint enables forward and backward movements, the CTr-joint enables elevation and depression of the leg, and the FTi-joint enables extension and flexion of the tibia of the leg. The yellow bars show swing phase while the other parts show stance phase. **(E)** Gait diagram showing a slow gait of AMOS. Black boxes indicate swing phase while white areas between them indicate stance phase. (**F**) A comparison of specific resistance of AMOS, with and without shark skin, during walking on carpet slopes. (**G**) A comparison of specific resistance of AMOS, with and without shark skin, during walking on PVC plastic flooring slopes. In case of without shark skin, default plastic parts on the belly made contact to the surface. We performed ten runs for each walking experiment. The error bars represent standard deviations. We encourage readers to also see Supplementary Movie 2 illustrating the tests.

Figure 2A shows the first setup of our locomotion tests. Figure 2B–E present AMOS walking up a slope. AMOS walked with a slow gait, with a low center of mass, driven by neural locomotion control^31^ (see the Methods section). With this walking behavior, all the legs swing (off the ground) and stance (on the ground) almost at the same time. Thus, the belly of AMOS touches the ground during the swing phase and stays above the ground with low ground clearance during the stance phase. An advantage of this walking behavior is that AMOS can rest on its belly during the swing phase. Thus, the motors of the legs do not need to produce high torque to carry the load (i.e., body weight). This also avoids unstable locomotion (i.e., tipping over or falling down) in case of leg damage^31^. In these tests, AMOS was equipped with standard rubber feet.

Figure 2F and G give a comparison of the specific resistance^32^ when AMOS, with and without shark skin on its belly, walked up different slopes having different surfaces. The specific resistance is the ratio between the consumed energy and the transferred gross weight times the distance traveled: 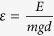, where *E* is energy, *mg* is the weight of AMOS (56.84N), and *d* is the distance traveled (here 1 m). The energy is estimated from: *E* = *IVt. I* is average electric current in amperes used by the motors of AMOS during walking 1 m. It is measured using the Zap 25 current sensor installed inside AMOS. *V* is voltage (here 5 V). *t* is time in seconds for the traveled distance. Low *ε* corresponds to highly energy-efficient walking. The results show that AMOS with shark skin successfully walked up 20° and 30° slopes covered by carpet as well as 15° and 20° slopes covered by PVC plastic flooring. In contrast, without shark skin (i.e., only a default smooth isotropic plastic material on its belly) AMOS failed to walk up the 30° carpet slope and the 20° PVC plastic flooring slope.

**Figure 3:**
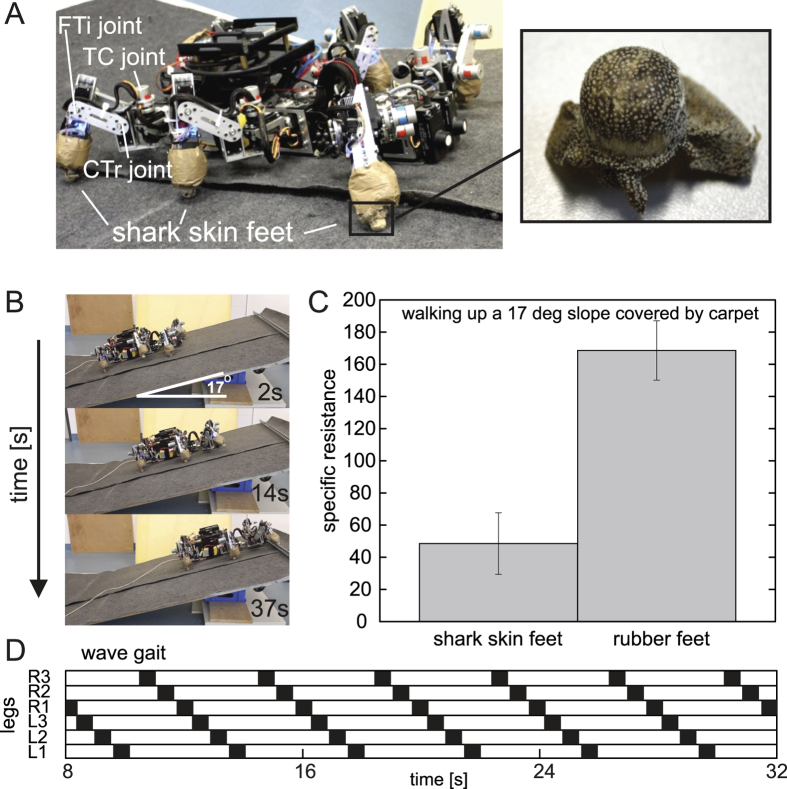
Testing locomotion of AMOS, with and without shark skin feet, on a slope. **(A)** AMOS with shark skin feet. (**B**) Snap shots of walking up a 17° slope covered by carpet where AMOS used the shark skin feet. (**C**) A comparison of specific resistance during walking with the rubber feet and the shark skin feet. (**D**) Gait diagram. Black bars show swing phase and white bars show stance phase. We performed five runs for each walking experiment. The error bars represent standard deviation. Note that the slope angle used here is the maximum angle that AMOS can walk up using its rubber feet. Based on this, average friction coefficient between the rubber feet and carpet is approximately 0.305. We refer to Fig. 1C for average friction coefficient between the shark skin feet and carpet. We encourage readers to also see Supplementary Movie 4 illustrating the tests.

Figure 3A and B show the second setup of our locomotion tests. Here, we let AMOS walk up a 17° carpet slope with the standard rubber feet and feet covered with shark skin in a typical wave gait. The angle of 17° was the maximum slope angle the robot could achieve with the standard rubber feet. With this wave gait, the belly of AMOS always stays above the ground (Fig. 3B and D); thereby locomotion cannot be enhanced by using shark skin installed on its belly as shown in the previous experiments. The shark skin feet were prepared from hydrated shark skin tightly pressed in a negative wooden form resembling the geometry of the robot feet. The shark skin was then dried for several days and remained stable in the robot foot geometry after removal from the form (Fig. 3A, right).

Figure 3C provides a comparison of the specific resistance when AMOS with shark skin feet and with standard rubber feet walked up the slope, respectively. The experimental result shows that using the shark skin feet leads to lower specific resistance, thereby more energy efficient walking, compared with the standard rubber feet. For a direct comparison of the specific resistance of the wave gait (Fig. 3D) in the second locomotion experiment and the slow gait (Fig. 2E) in the first locomotion experiment on a 17° slope covered by carpet, we linearly extrapolate the specific resistance data with shark skin shown in Fig. 2F. The result shows that the specific resistance of the slow gait is approximately 70% (specific resistance of ≈85) higher than that obtained from the wave gait (specific resistance of ≈50, Fig. 3C). This is because the walking speed of the slow gait is much slower that the wave gait; AMOS therefore requires in total more energy to walk, with the slow gait, up the slope for the given distance. However, the shark skin feet are more quickly destroyed from the wave gait than the shark skin on the belly with the slow gait.

For a comparison, we also used a stainless steel rasp with friction isotropy. This material interlocks with the carpet in both directions. Thus, the high-friction isotropic material not only prevents AMOS from sliding or slipping, while walking on a slope covered by carpet, but also makes it difficult to release or disengage its belly or feet from the surface in order to move forward. As a consequence, AMOS gets stuck on the surface (see Supplementary Movie 3). Average friction coefficient between the steel rasp surface and the carpet is approximately 1.6 in both directions. In contrast, shark skin’s asymmetric profile, like a sloped array of spines, generates strong mechanical interlocking with the surface in one direction (rostral direction) and almost no mechanical interlocking in the other direction (caudal direction); thereby enhancing locomotion efficiency.

### Tribological characterization of shark skin

**Figure 4:**
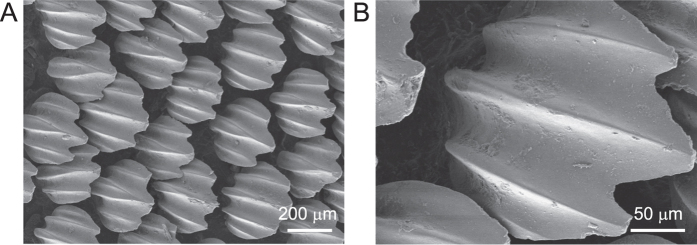
Shark skin. (**A**) Intact shark skin. (**B**) An individual scale.

In addition to the locomotion experiments with the walking robot we also performed friction experiments with shark skin (Fig. 4), in order to understand its friction behavior under different experimental conditions. Friction experiments were performed with dry shark skin under dry conditions and with fresh shark skin under water (wet condition). Moreover, friction experiments were performed on four different rough substrates, with two different applied normal loads, and in different sliding directions (see the Methods section).

**Figure 5:**
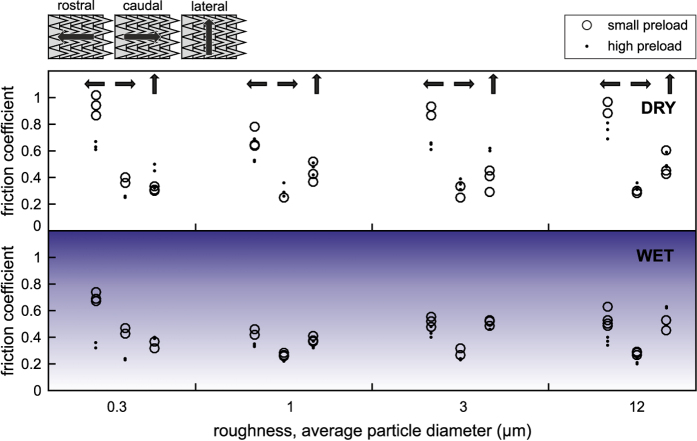
Measured friction coefficients of shark skin under dry and wet conditions with small applied preload and high preload in different directions. The friction coefficients with small applied preload and high preload are shown in open circles and points, respectively. Rostral, caudal, and lateral directions in dependence of the substrate roughness were tested. Schematics above the graph illustrate the sliding direction in relation to the shark skin orientation with its anisotropic arrangement of denticles.

Figure 5 shows that friction in the rostral direction (sliding against the denticles of the shark skin) was always higher than when sliding in caudal direction (i.e., sliding along the denticles). This effect occurred independently of the applied normal load, the substrate roughness, and the measurement condition (wet or dry). Due to this systematic frictional anisotropy between rostral and caudal direction, data of the four rough substrates and the two normal loads were pooled together.

**Figure 6:**
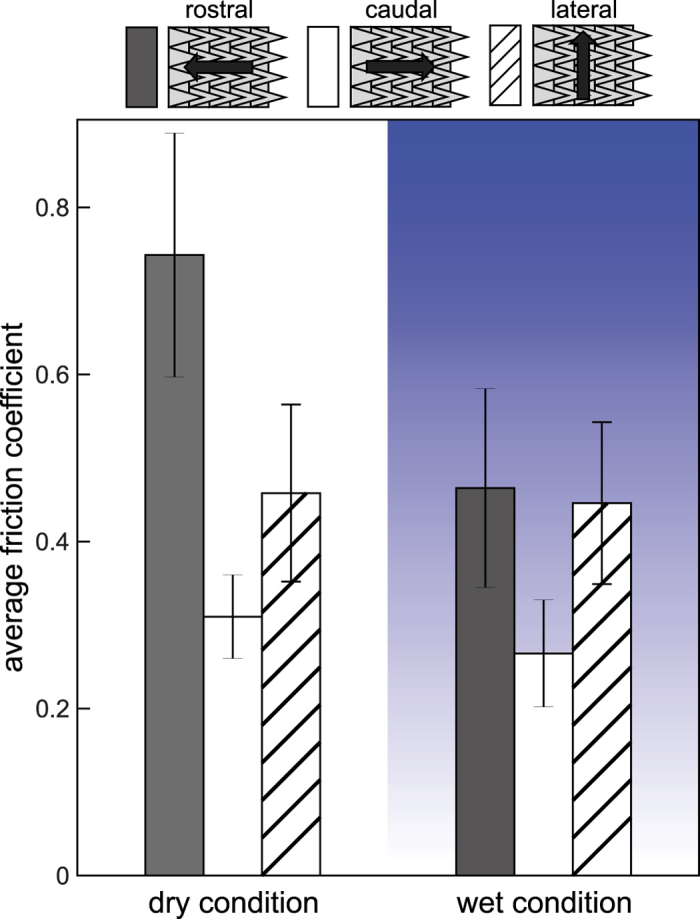
Average friction coefficients of shark skin under dry and wet conditions measured for different sliding directions. Three directions (rostral, caudal, and lateral) were tested. Schematics above the graph illustrate the sliding direction in relation to the shark skin orientation with its anisotropic arrangement of denticles.

Figure 6 shows the averaged (over all substrates and normal loads) friction coefficients under dry and wet conditions for the three different sliding directions. Absolute values of friction coefficients were in the range from about 0.2 to about 0.9. Table 1 shows the statistical results of the pairwise multiple comparison obtained by the Holm-Sidak post-hoc test, which has been performed after a Two Way ANOVA (see the Methods section). Friction in rostral direction was significantly higher than friction in caudal direction, with dry and wet conditions. The anisotropy, i.e. the ratio between friction coefficients in rostral and caudal direction, is almost 2.4 for dry conditions and about 1.7 for wet conditions. In the dry state, lateral friction is between that measured in rostral and caudal directions and was significantly different from both. Friction in dry and wet states was very similar for the caudal and lateral directions, but in the rostral direction, friction in the wet state was significantly lower if compared to the dry state.

**Figure 7:**
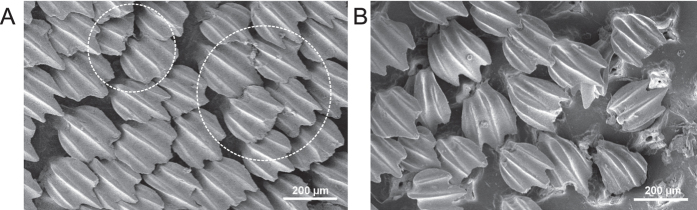
The abrasion of shark skin samples during friction tests. (**A**) In the wet state (≈7.1 kPa) some denticles show abrasion wear as indicated by dashed circles. (**B**) In the dry state (≈8.8 kPa) individual denticles were teared out of the skin.

Although there was no systematic influence of the applied normal load on the frictional anisotropy, higher applied normal load led to partial damage of the shark skin. Figure 7 shows shark skin in wet (A) and dry (B) state after multiple measurements at the higher applied normal load, which was ≈7.0 kPa in the wet state and ≈8.8 kPa in the dry state, respectively (see the Methods section). The high load corresponds approximately to the normal load (AMOS’ weight) applied to the shark skin in the first robot experiment. In the wet state, abrasion was present on individual denticles and some were broken, but remained attached to the skin (Fig. 7A). In the dry state, individual denticles were torn out of the skin at that normal load (Fig. 7B). For the smaller applied normal load, which was ≈1.6 kPa in the wet state and ≈2.2 kPa in the dry state respectively, no obvious damage occurred.

## Discussion

In this study, we showed how the usage of shark skin effects to enhance grip and locomotion of a walking robot. Two different walking gaits generated by a neural locomotion controller were used for robot experiments: A slow gait where all the legs swing and stance almost at the same time and a wave gait where the right legs move in succession from the back to the front and followed by the left legs. For the slow gait, the skin was installed on the belly of the robot since the belly touches during a swing phase while for the wave gait the skin was used to cover normal robot feet since the belly stays above the ground. The robot experimental results reveal that using an anisotropic material (dry shark skin) can generate strong grip and energy-efficient locomotion of the robot for walking up different slope angles with different surfaces (laminated plywood, PVC plastic flooring, and carpet) without high control effort. While the robot experiments were performed using shark skin operating in dry conditions, in principle the anisotropy effect of shark skin can be also utilized for a robot operating in humid or wet conditions and even under water since the effects in both states were estimated in our friction experiments.

The strategy of exploiting material properties to achieve complex behaviors without complex control is considered part of morphological computation^18^,^19^. Based on the principles of morphological computation, most research focuses mainly on exploiting the property of soft/flexible materials for flexible and robust robot locomotion^30^,^31^,^32^,^33^,^34^,^35^,^36^. Our study here complements the morphological computation principles by using a passive anisotropic scale-like material and its interaction with the environment for energy-efficient locomotion and operational range expansion. The material property generates frictional anisotropy and mechanical interlocking between surfaces without the need for any sensory feedback, modifying our existing locomotion control, or even redesigning our robot structures. This makes our solution simple and cheap. Our approach is also different from other developments which require complex motion control or/and special robot structures (e.g., active scales) to achieve frictional anisotropy for efficient locomotion^26^,^37^,^38^.

To support the frictional anisotropy observed in our robot experiments, friction measurements with shark skin on the different rough substrates were performed. The observed frictional anisotropy of shark skin is most likely an effect solely due to the anisotropic arrangement of individual denticles on the skin, which allows mechanical interlocking with surface asperities in one direction and not in the opposite direction. These findings are in agreement with the recent observations of frictional anisotropy supporting locomotion in some snake species, which was also attributed to the anisotropic geometry of scale microstructures^39^,^40^,^41^,^42^.

In general, anisotropy of surface micro- and nanostructures in biological surfaces are wide-spread and serve a variety of different functions^6^ such as locomotion, fluid/particle transportation, fixation, etc. Although we have observed promising results employing shark skin to enhance locomotion efficiency of our walking robot, we have to stress that we do not propose employing shark skin in future industrial robotic applications. In fact, using shark skin we were able to learn more on the design constraints of potential artificial surfaces with anisotropic friction. For example, obvious damage in the form of torn out individual denticles has been observed at sufficiently high applied normal load on dry skin. Hydrated skin, at similar normal load, did not show torn out denticles. Individual denticles are embedded into the skin in socket-like structures consisting of a collagen matrix^43^. In hydrated skin these sockets are flexible and may act as elastic joint-like elements, which allow individual denticles a certain degree of freedom to bend without release from the skin. In dry skin, however, individual denticles may sustain a higher overall load but at a certain threshold denticles may spontaneously fail and be released from the skin. This also seems to be a reasonable explanation why friction in the rostral direction is lower for the wet skin compared to the dry skin. An “overload protection” mechanism, like the joint-like fixation of stiff structures embedded in the soft matrix, seems to be a general principle in nature. It can be found, for example, in adhesive tarsal setae of insects^44^, in hooks covering the diaspore of the cleaver plant *Galium aparine*^45^, and in feeding tools of e.g. copepods, which consist of opal teeth connected to the mandible via a rubber-like bearing^46^. Such joint-like elastic elements generally reduce stress concentrations^47^ and may allow structures to go round the applied load avoiding mechanical failure.

Taken together this work suggests that employing an anisotropic scale-like material in a walking robot can effectively enhance locomotion efficiency and even increase its field of action without further need for complex control and sensory feedback of the locomotion pattern. However, such a biological anisotropic scale-like material is still less robust compared to artificial materials (like rubber) since it would be prone to be easily damaged after several runs. Thus, in the future, we will design synthetic microstructured surfaces that mimic the investigated tribological characterization of shark skin, for prolonged usage with an efficient overload protection mechanism in real (robotic) applications. Furthermore, we will explore the use of such material not only to enhance locomotion efficiency as shown here but also further to achieve adaptive locomotion on a rough surface slope. In this case, learning mechanisms^48^ will be applied to allow a robot to adapt its walking behavior online to deal with leg damage while the shark skin-inspired material will prevent the robot from slipping or sliding backward. We believe that this future strategy, which exploits the synergy of adaptive control and a passive anisotropic shark skin-inspired material, will open up the opportunity of implementing adaptive, energy-efficient, and robust locomotion behavior of robots for navigating and moving in difficult terrain.

## Methods

All methods and experiments reported herein were carried out in accordance with the approved guidelines.

### Sample preparation

For our experiments, skin of a dead porbeagle (*Lamna nasus*), which belongs to the animal collection of the Zoological Institute of Kiel University, Germany, was used. A skin sheet was cut out from the ventral abdomen of the shark and was kept deep-frozen at −20 °C. Four samples 20 × 40 mm were sawn out from the frozen skin. Fleshy parts of the skin were removed. Two samples were pinned along the perimeter on a wood block and dried under environmental conditions. Other two samples were stored in 2% diethylene glycol before the experiment. Replicas of a sleigh made of wood with polishing paper having different roughness (0.3, 1, 3, 12 *μ*m) glued to the bottom of the sleigh were produced from Spurr epoxy resin^49^. To avoid possible edge effects during friction force measurements all edges of the sleighs were rounded off. The bottom area of the sleigh was 15 × 15 mm. Negative replicas were produced using dental wax (President light body, Colthéne/Whaledent AG, Altstätten, Switzerland).

### Friction measurements

**Figure 8:**
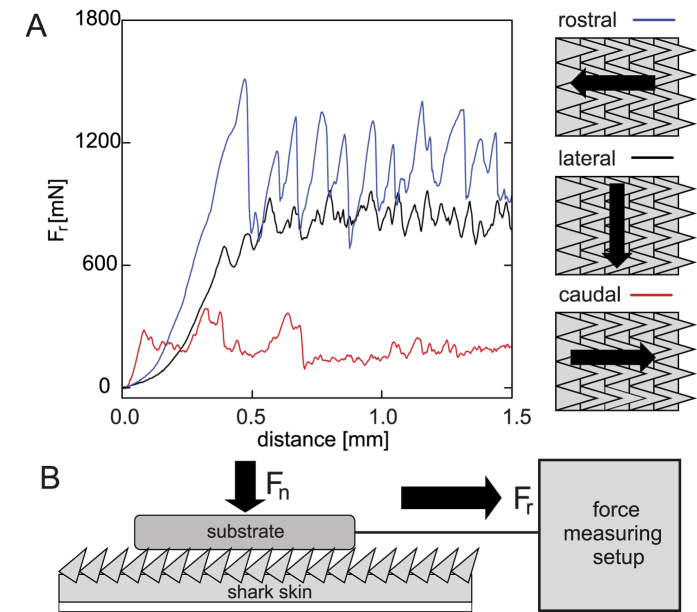
Experimental setup for friction measurements. (**A**) Typical force-distance curves (obtained for dry shark skin) measured in three different directions on the rough substrate with an average particle diameter of 12 *μ*m. **(B)** Friction measurements performed by pulling substrates with different roughness over a shark skin sample. Normal load was applied with defined weights.

An example of friction measurement and the scheme of an experimental setup for determination of the friction coefficient of the shark skin in different directions on different roughness are presented in Fig. 8A and B, respectively. The shark skin samples were glued on a glass slide. Positive replicas of polishing papers with 0.3 *μ*m, 1 *μ*m, 3 *μ*m and 12 *μ*m particle size (Buehler, Lake Bluff, IL, USA) were used as a rough counterpart in friction measurements. Spurr replica of sleigh with different roughness was placed on the shark skin. Different loadings were set with 50 g and 200 g weights placed on the sleighs. Spurr sleigh was linked to a force transducer (FORT1000, World Precision Instruments, Sarasota, FL, USA) using a non-stretchable nylon cord. The force transducer was mounted on a motorized manipulator, which pulled the sleigh 10 mm with 2.5 mm/s velocity. The signal from the force transducer was acquired by Biopac System (Biopac Systems Inc., Goleta, CA, USA) and recorded using AcqKnowledge 3.7.0. software (Biopac Systems Inc., Goleta, CA, USA). The friction was measured in three different directions: caudal (along the denticles), rostral (against the denticles), and lateral; on different roughness (0.3 *μ*m, 1 *μ*m, 3 *μ*m and 12 *μ*m); at two different loadings (50 g and 200 g); in the sea water (the water from Baltic Sea, fresh defrosted shark skin) and in a dry state. For each parameter combination, the friction measurements were performed at least three times. After each set of three measurements it was checked whether the skin was damaged and a new location was taken for further measurements, if needed. Friction coefficient *μ* = *F*_*r*_/*F*_*n*_ was calculated using the highest pulling force *F*_*r*_ within the first 0.5 mm pulling distance. The initial region of the force-distance curve corresponds to both the straightening of the thread connecting the substrate and the force sensor and slight rotations of the substrate on the shark skin. However, we could verify that the onset of sliding motion was within 0.5 mm. All the measurements were performed under normal conditions (room temperature, 40% humidity).

### Microscopy

Dried samples were sputter coated with ≈10 nm of Au/Pd. Imaging was performed with a Hitachi TM3000 tabletop scanning electron microscope (Hitachi High-Technologies Corp., Tokyo, Japan) at an accelerating voltage of 3 kV.

### Statistics

Since observed frictional anisotropy was independent of the substrate roughness and applied normal load the data were pooled correspondingly. In order to test significant differences for the different sliding directions both in the dry and wet state a Two-Way ANOVA has been performed. Although both assumptions of normal distribution and homoscedasticity were violated, the ANOVA was, however, performed. Since sample sizes in each group were approximately the same^50^,^51^ and the ratio of the largest group variance to the smallest was smaller than 10^52^, the ANOVA was considered to be sufficiently robust. Moreover, the Holm-Sidak multiple comparison post-hoc test was performed afterwards, which does not assume homoscedasticity. Statistical analysis has been performed using SigmaPlot 12.5 (Systat Software, Inc, San Jose, California, USA).

### Neural locomotion control

Neural control for locomotion generation of the bio-inspired walking machine AMOS was developed in earlier work^31^. Here we used it without any modification and sensory feedback for our robot experiments. The control consists of three neural modules: Central pattern generator (CPG)-based control module with neuromodulation, neural CPG postprocessing module and neural motor control module. The CPG-based control module generates different periodic signals to obtain different gaits. The postprocessing module shapes the CPG periodic signals to obtain smooth leg movements. The motor control module consists of two additional different networks [phase switching network (PSN) and velocity regulating networks (VRNs)] for controlling walking direction (forward/backward and turning). The final outputs from the motor control module are transmitted through delay lines to all leg joints of AMOS. All neurons of the locomotion control network are modeled as discrete-time non-spiking neurons. They are updated with a frequency of approximately 27 Hz. The activity *a*_*i*_ of each neuron develops according to:


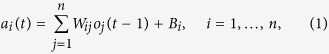


where *n* denotes the number of units, *B*_*i*_ an internal bias term or a stationary input to neuron *i, W*_*ij*_ the synaptic strength of the connection from neuron *j* to neuron *i*. The output *o*_*i*_ of all neurons of the network is calculated by using the hyperbolic tangent (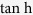) transfer function, i.e., *o*_*i*_ = tan h(*a*_*i*_), ∈[−1, 1], except for the CPG postprocessing neurons using a step function, the motor neurons using piecewise linear transfer functions, and neurons in searching and elevation control using a linear transfer function. The complete description of the locomotion control network can be seen in our previous work^31^.

### Bio-inspired walking machine

The six-legged walking machine AMOS is a biologically inspired hardware platform. It consists of six identical legs, each having three joints (three degrees of freedom): the thoraco-coxal (TC-) joint enables forward (+) and backward (−) movements, the coxo-trochanteral (CTr-) joint enables elevation (+) and depression (−) of the leg, and the femoro-tibial (FTi-) joint enables extension (+) and flexion (−) of the tibia. The morphology of this multi-jointed leg is modeled on a cockroach leg but the tarsal segments are ignored. All joints are driven by standard servomotors. The walking machine has all in all 19 motors and 32 sensors. We use a Multi-Servo IO-Board (MBoard) to digitize all sensory input signals and generate a pulse-width-modulated signal to control servomotor position. For the robot walking experiments in this study, the MBoard was connected to a personal computer on which the neural locomotion controller was implemented. The update frequency was 27 Hz. Electrical power supply was provided by batteries: one 11.1 V lithium polymer 3200 mAh for all servomotors and two 11.1 V lithium polymers 910 mAh for the electronic board (MBoard) and all sensors (see ref. 31 for more details).

## Additional Information

**How to cite this article**: Manoonpong, P. *et al*. Enhanced Locomotion Efficiency of a Bio-inspired Walking Robot using Contact Surfaces with Frictional Anisotropy. *Sci. Rep.* **6**, 39455; doi: 10.1038/srep39455 (2016).

**Publisher's note:** Springer Nature remains neutral with regard to jurisdictional claims in published maps and institutional affiliations.

## Supplementary Material

Supplementary Video 1

Supplementary Video 2

Supplementary Video 3

Supplementary Video 4

Supplementary Video 5

Supplementary Information

## Figures and Tables

**Table 1:** Statistical significance of differences between friction coefficients measured under different experimental conditions (Two Way ANOVA and a Holm-Sidak multiple comparison post-hoc test).

Condition	Sliding direction	Significant difference
dry	rostral versus caudal	yes (p < 0.001)
dry	rostral versus lateral	yes (p < 0.001)
dry	caudal versus lateral	yes (p < 0.001)
wet	rostral versus caudal	yes (p < 0.001)
wet	rostral versus lateral	no (p = 0.548)
wet	caudal versus lateral	yes (p < 0.001)
dry versus wet	rostral	yes (p < 0.001)
dry versus wet	caudal	no (p = 0.198)
dry versus wet	lateral	no (p < 0.705)
