# Supplementary material for: Enhanced Locomotion Efficiency of a Bio-inspired Walking Robot using Contact Surfaces with Frictional Anisotropy
Source: Sci Rep. 2016 Dec 23;6:39455. doi: 10.1038/srep39455 (PMC5180203; doi:10.1038/srep39455)
Supplement: Supplementary Information [file srep39455-s6.pdf]

Supplementary information accompanying the manuscript
Enhanced Locomotion Efficiency of a Bio-inspired Walking Robot using Contact Surfaces with Frictional Anisotropy

Poramate Manoonpong[1,2,*], Dennis Petersen[3], Alexander Kovalev[3], Florentin Wörgötter[2], Stanislav Gorb[3], Marlene Spinner[3], Lars Heepe[3,4]

[1)]Embodied AI and Neurorobotics Lab, Centre for BioRobotics,
The Mærsk Mc-Kinney Møller Institute,
University of Southern Denmark,
DK-5230 Odense M, Denmark
[2)]Bernstein Center for Computational Neuroscience (BCCN),
The Third Institute of Physics,
Georg-August-Universität Göttingen,
D-37077 Göttingen, Germany
[3)]Department of Functional Morphology and Biomechanics,
Zoological Institute,
Kiel University,
D-24118 Kiel, Germany
[4)]Mads Clausen Institute, University of Southern Denmark,
DK-6400 Sønderborg, Denmark

[*]Correspondence: poma@mmmi.sdu.dk

Testing Seal Skin

We performed two tests to investigate the property of seal skin: 1) Testing grip of AMOS, with seal skin, on different surfaces and 2) testing locomotion of AMOS, with seal skin on its belly, on different slopes with different surfaces.

Supplementary Figure 1 shows that seal skin has frictional anisotropy as shark skin (cf. Fig. 1C of the main manuscript text). Comparing average static friction coefficients of seal skin and shark skin, seal skin has less friction than shark skin on rough surfaces (e.g., PVC plastic flooring and carpet) but more friction on smooth surface (e.g., laminated plywood).

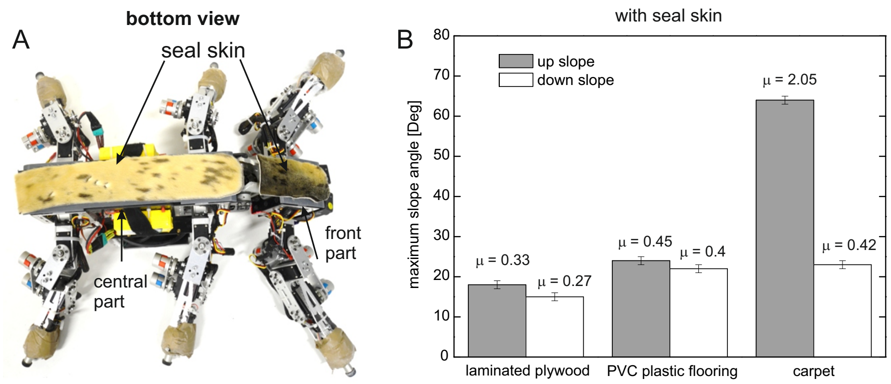

Supplementary Figure 1: **Testing grip of AMOS, with seal skin, on different surfaces. (A)** Two pieces of seal skin installed at the front and central parts of the hexapod walking robot AMOS. The seal skin at the front part has a size of 6 cm wide and 13 cm long while another one at the central part is 6 cm wide and 31 cm long. **(B)** A comparison of maximum slope angles before AMOS, with seal skin, started to slip on three different surfaces in up slope and down slope directions (cf. Fig. 1B of the main manuscript text). Average static friction coefficients between seal skin and the surface for all tests are calculated from $tanh(\theta_{max})$ and depicted on top of the columns. We performed ten runs for each surface. The error bars represent standard deviation.

Supplementary Figure 2 shows average specific resistance during locomotion using seal skin. Comparing specific resistance of seal skin and shark skin, average specific resistance values of locomotion using seal skin are approximately 135 and 417 (see Supplementary Figure 2A) and shark skin are approximately 99 and 156 (see Fig. 2F of the main manuscript text) for 20 deg and 30 deg slopes covered by carpet, respectively. For PVC plastic flooring slopes, average specific resistance values of locomotion using seal skin are approximately 140 and 220 (see Supplementary Figure 2B) and shark skin are approximately 129 and 207 (see Fig. 2G of the manuscript) for 15 deg and 20 deg slopes, respectively. This shows that locomotion using shark skin is more energy efficient than seal skin.

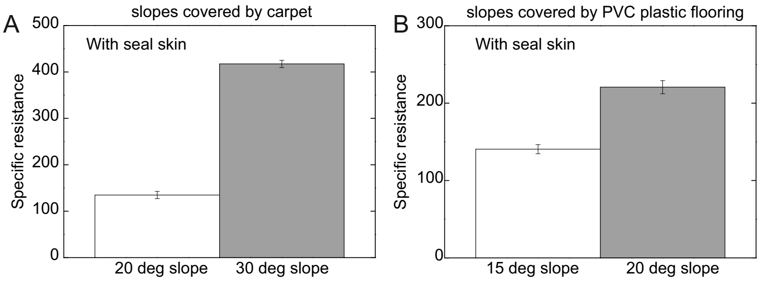

Supplementary Figure 2: **Testing locomotion of AMOS, with seal skin on its belly, on different slopes with different surfaces.** **(A)** A comparison of specific resistance of AMOS, with seal skin, during walking on carpet slopes. **(B)** A comparison of specific resistance of AMOS, with seal skin, during walking on PVC plastic flooring slopes. We performed ten runs for each walking experiment. The error bars represent standard deviations. We encourage readers to also see Supplementary Movie 5 illustrating the tests.

Supplemental Movie Legends

Supplementary Movie 1: **Testing grip of the hexapod robot AMOS, with shark skin, on a rough surface**. The hexapod robot AMOS can strongly grip a rough surface (e.g., carpet) without control due to mechanical interlocking between shark skin on its belly and the surface.
(http://www.manoonpong.com/nature/SupplementaryMovie1.mov)

Supplementary Movie 2: **Testing the performance of shark skin for walking up different slopes with different surfaces**. Walking performance of the hexapod robot AMOS, with and without shark skin on the belly, on different slopes with different surfaces.
(http://www.manoonpong.com/nature/SupplementaryMovie2.mov)

Supplementary Movie 3: **Testing an isotropic material for walking up carpet slopes**. Locomotion of the hexapod robot AMOS with an isotropic material with spiky surface (e.g., a stainless steel rasp) on carpet slopes.
(http://www.manoonpong.com/nature/SupplementaryMovie3.mov)

Supplementary Movie 4: **Testing the performance of shark skin feet and rubber feet for walking up a 17 degree slope covered by carpet**. Walking performance of the hexapod robot AMOS, with shark skin feet and with rubber feet, on the 17 degree carpet slope.
(http://www.manoonpong.com/nature/SupplementaryMovie4.mov)

Supplementary Movie 5: **Testing seal skin for walking up different slopes with different surfaces**. Locomotion of the hexapod robot AMOS, with seal skin on the belly, on different slopes covered by carpet and PVC plastic flooring.
(http://www.manoonpong.com/nature/SupplementaryMovie5.mov)
